# exTra Mini-review Series on ECP in Transplantation

**DOI:** 10.1097/TXD.0000000000001860

**Published:** 2025-09-02

**Authors:** James A. Hutchinson, Edward K. Geissler

**Affiliations:** 1 Department of Surgery, University Hospital Regensburg, Regensburg, Germany.

Although early transplant outcomes are now generally excellent, modern practice still faces significant unmet challenges, especially long-term immunosuppression-related toxicity and chronic decline of transplant function. Innovative strategies are needed to lessen our reliance upon conventional immunosuppression and better control nonresolving inflammation. Extracorporeal photopheresis (ECP) is widely established as a personalized, adjunct immunosuppressive therapy in heart and lung transplantation.^1^ By contrast, ECP has not been commonly adopted in kidney and liver transplantation.^2^ The exTra project (extra-horizon.eu) is a new European Union-funded network that aims to extend applications of ECP in solid organ transplantation through a deeper mechanistic understanding of its immunological actions. exTra brings together European experts in clinical transplantation, immunology, pharmaceutical development, computational biology, and medical device manufacturing to address basic and translational research questions. Specifically, the network is studying possible roles for ECP in 3 main areas—namely, regulating T cell alloimmunity, controlling antibody-mediated rejection, and promoting tissue repair to prevent allograft fibrosis.^3^ Through a coordinated, interdisciplinary effort, the exTra project is contributing to a solid scientific and technological foundation for future use of ECP in solid organ transplantation.

ECP is a clinical technique for generating apoptotic leucocytes that are readministered to patients as an immunoregulatory therapy. Signals from apoptotic cells are critical for maintaining peripheral T cell tolerance and directing tissue remodeling. ECP allows us to exploit these fundamental mechanisms to regulate alloimmunity and resolve allograft damage. However, many questions about implementing ECP in transplant recipients remain unanswered: What is an optimal cell dose? What is the optimal treatment interval? Why do some patients respond and others not? These questions can only be addressed through a deeper immunological understanding of ECP and the development of clinical assays to monitor treatment responses.

The current issue of *Transplantation Direct* contains a series of mini-reviews from exTra participants that explain the scientific background of the network, highlighting points of consensus and controversy in the field. Alemanno^4^ describes current and future applications of ECP in lung transplantation, while Nicoli^5^ examines its possible uses in kidney transplantation. Both articles emphasize the potential applications of ECP in preventing or treating antibody-mediated rejection by suppressing circulating donor-specific antibody levels. Nogueira^6^ seeks a mechanistic explanation for the specific reduction of circulating donor-specific antibody in ECP-treated transplant recipients, which might include suppression of follicular helper T cell activation, direct effects of apoptic leucocytes on B cells or plasma cells, and generation of regulatory B responses.

There is an emerging agreement that the primary effects of ECP depend on apoptotic cells modifying macrophages and dendritic cells, which then regulate effector responses. Stępień^7^ explores cell death pathways triggered by ECP in leucocytes, explaining how noninflammatory cell death counterbalances transplant-related injury at a molecular level.^7^ Garcia-Almeida^8^ expands upon the immunomodulatory properties of apoptotic cells with a comprehensive review of soluble factors present in ECP products. Arella^9^ discusses the phenotypic and functional changes that occur in macrophages and dendritic cells after exposure to apoptotic lymphocytes, which confer them with T cell-suppressive and tissue-reparative properties. Tocco^10^ further explores how apoptotic cell exposure might alter the behavior of mononuclear phagocytes by antagonizing tissue injury–related signals that would otherwise induce trained immunity.^10^

Successfully translating new indications for ECP into clinical practice requires preclinical demonstration of efficacy,^11^ reliable biomarkers to guide treatment decisions,^12^ and a pathway to regulatory approval. Morgado^13^ describes current methods for studying ECP in animal models and their relevance to clinical practice. Starting from a current understanding of ECP’s therapeutic actions, Veltman^14^ collates a list of biomarkers associated with favorable treatment responses in patients. Finally, Parsonidis explains how ECP fits into the broader landscape of immune regulatory cell-based therapies.^15^

Altogether, these mini-reviews give a state-of-the-art overview of current and future applications of ECP in solid organ transplantation, stressing the importance of a solid mechanistic understanding of ECP in the design and evaluation of future clinical trials. An emerging theme is the importance of early intervention with ECP, including as induction therapy, rather than its current place as a treatment of last resort. This series of articles also highlights the context-dependent effects of ECP, which reflect the diverse roles of macrophages and dendritic cells in various aspects of transplant pathology. Finally, by showing how ECP powerfully suppresses alloimmune responses, these mini-reviews remind us that autologous apoptotic cell clearance is vital to establishing and maintaining peripheral tolerance.
